# Three-input logic gate based on DNA strand displacement reaction

**DOI:** 10.1038/s41598-023-42383-9

**Published:** 2023-09-14

**Authors:** M. A. Jingjing

**Affiliations:** School of Statistics, Shanxi University of Finance and Economy, Taiyuan, 030000 China

**Keywords:** Biochemistry, Nanoscience and technology

## Abstract

In this paper, three kinds of three-input logic gates are designed based on DNA strand displacement reaction, which are three-input OR logic gate, three-input AND logic gate, and three-input MAJORITY logic gate. The logic gates designed in this paper takes different DNA strands as input and fluorescence signals as output. The biochemical experimental results verify my designs. The results show that DNA strand displacement technology has important application value in DNA computing, especially in the construction of DNA molecular logic gates.

## Introduction

In 1953, Watson and Crick discovered the double helix structure of DNA, which opened the era of Molecular Biology, and made the research of Genetics go deep into the molecular level. In 1959, Feynman^1^ proposed the idea of molecular computing, he suggested to use single molecule or atom to construct computer components. In 1994, Adleman^2^ solved a Hamiltonian Path problem with seven vertices through a biochemical experiment, and proved the ability of DNA computing in solving complex Mathematical problems. After that, great progress has been made in the theoretical and experimental aspects of DNA computing^3–8^. Compared with the traditional electronic computer, DNA computing has many advantages, such as large amount of information storage, high parallelism, low energy consumption. At the same time, the combination of DNA computing with the rapid development of Molecular Biological technology and Nanotechnology is bound to get greater development.

DNA strand displacement technology is a new technology in recent years. The existing studies show that DNA strand displacement technology has applications in the construction of DNA molecular logic gates^9^, biosensors^10^, nanorobotics^11^, and in the diagnosis and treatment of diseases such as molecular detection^12^, molecular drug loading^13–15^, and so on.

Qian constructed a complex large-scale cascade circuit^16^ by using DNA strand displacement technology, and realized four bit square root logic circuit^17^ and neural network^18^. Li realized 3-input logic gate and complex cascade circuit by using circular DNA molecule and DNA strand displacement technology^19^. Yanfeng constructed a variety of reusable combinational logic gates based on DNA strand displacement technology, and carried out simulation verification with visual DSD software^20^. Wei designed and experimentally realized a three-input majority gate based on DNA strand displacement^21^.

In this paper, three kinds of three-input logic gates are designed based on DNA strand displacement reaction, which are three-input OR logic gate, three-input AND logic gate, and three-input MAJORITY logic gate. The logic gates designed takes different DNA strands as input and fluorescence signals as output. The biochemical experimental results verify my design. The results show that DNA strand displacement technology has important application value in DNA computing, especially in the construction of DNA molecular logic gates.

## Materials and methods

### Design principles of logic gates based on DNA strand displacement reaction

**Figure 1:**
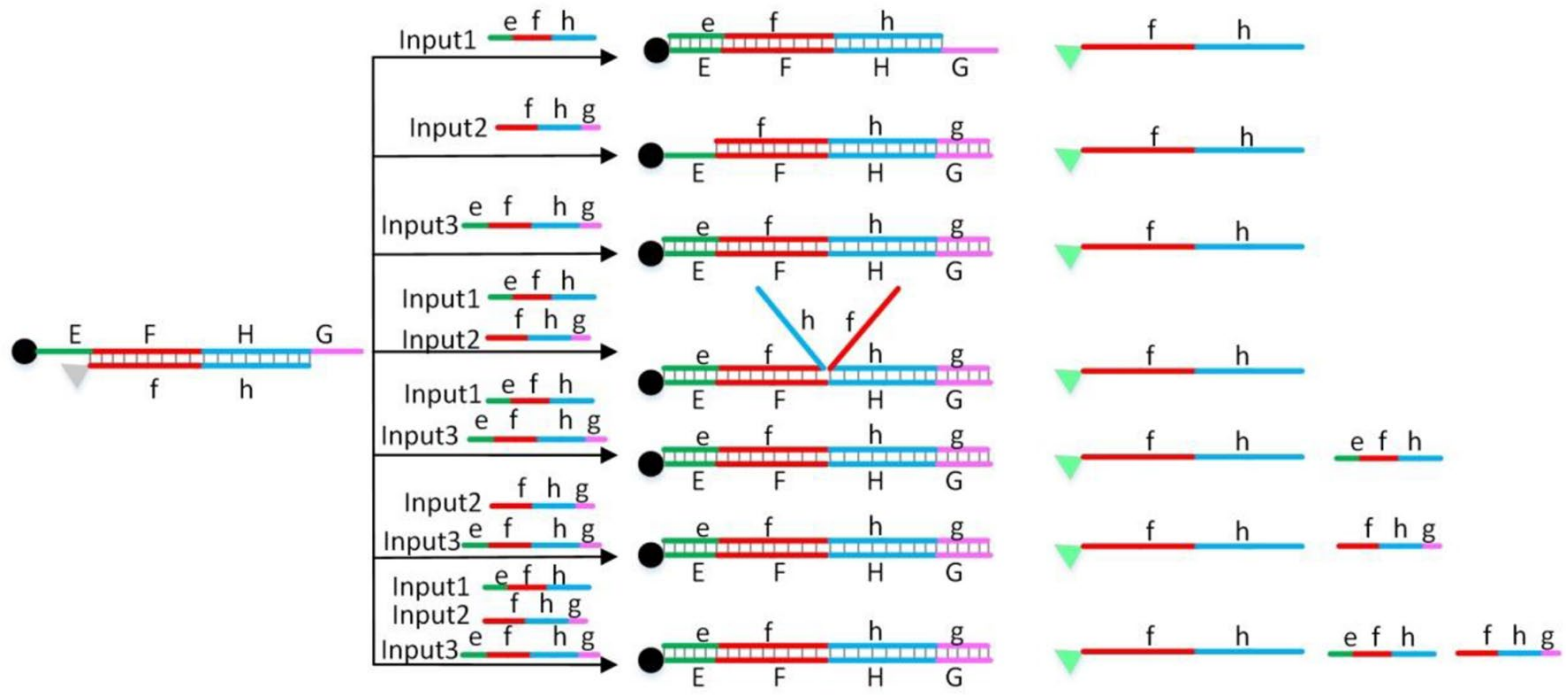
Three-input OR logic gate.

In this paper, as shown in Fig. 1, different color line segments are used to represent different DNA sequences, complementary sequences are represented by line segments of the same color, and the names of two complementary sequences are represented by upper-case and lower-case letters, respectively. For example, in Fig. 1, two red parallel lines represent two complementary sequences, which can be hybridized to form a double helix. The upper-case F and lower-case f are used to represent the names of the two complementary sequences, respectively. In this paper, as shown in Fig. 1, BHQ1 quencher is represented by small black circles, fluorescent groups are represented by small triangles, quenched fluorescent groups are represented by small gray triangles, and non-quenched fluorescent groups are represented by small green triangles.

Figure 1 shows the principle of the three-input OR logic gate based on DNA strand displacement reaction. In the initial state, that is, without any input, the 3-terminal modified BHQ1 single DNA strand EFHG can hybridize with the 5-terminal modified FAM single DNA strand fh to form a double helix. The fluorescence signal of FAM is quenched due to the close distance between BHQ1 and FAM fluorescent group. As shown in Fig. 1, I designed three kinds of DNA input strands respectively: INPUT1, INPUT2, INPUT3. INPUT1 is efh, INPUT2 is fhg, INPUT3 is efhg. According to the principle of DNA strand displacement reaction, three kinds of input strands can replace fh and hybridize with EFHG to release fh labeled by fluorescent group. After the fh is released, its fluorescent group is far away from the quencher, so its fluorescent signal will increase. As shown in Fig. 1, when one or two or three of the three input strands are added, the fluorescence signal will rise. Therefore, a three-input OR logic gate is formed with three input strands efh, fhg and efhg as input and fluorescence signal as output.

**Figure 2:**
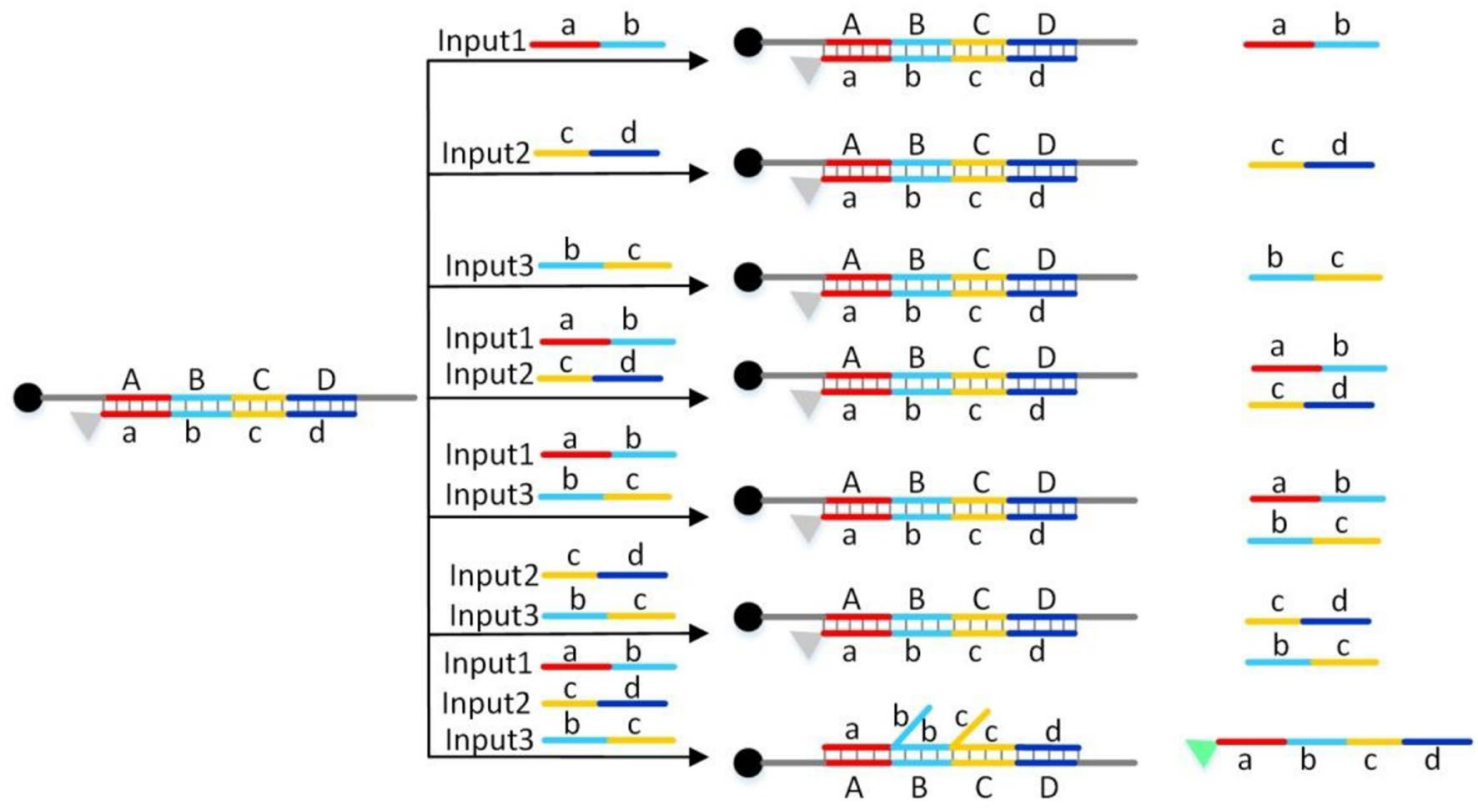
Three-input AND logic gate.

Figure 2 shows the principle of the three-input AND logic gate based on DNA strand displacement reaction. In the initial state, that is, without any input, the 3-terminal modified BHQ1 single DNA strand ABCD can hybridize with the 5-terminal modified FAM single DNA strand abcd to form a double helix. The fluorescence signal of FAM is quenched due to the close distance between BHQ1 and FAM fluorescent group. As shown in Fig. 2, I designed three kinds of DNA input strands respectively: INPUT1, INPUT2, INPUT3. INPUT1 is ab, INPUT2 is cd, INPUT3 is bc. According to the principle of DNA strand displacement reaction, when any one or two of the three input strands are added, the input strands can not displace the abcd strand, so the ABCD strand and the abcd strand are still hybridized, and the fluorescence signal of FAM is still quenched. Only when three input strands are added at the same time, ab, bc and cd could displace abcd, hybridize with ABCD to form double helix and release abcd. After the release of abcd, the fluorescent group is far away from the quencher, so the fluorescence signal will increase. As shown in Fig. 2, a three-input AND logic gate is formed with three input strands ab, bc and cd as input and fluorescent signal as output.

**Figure 3:**
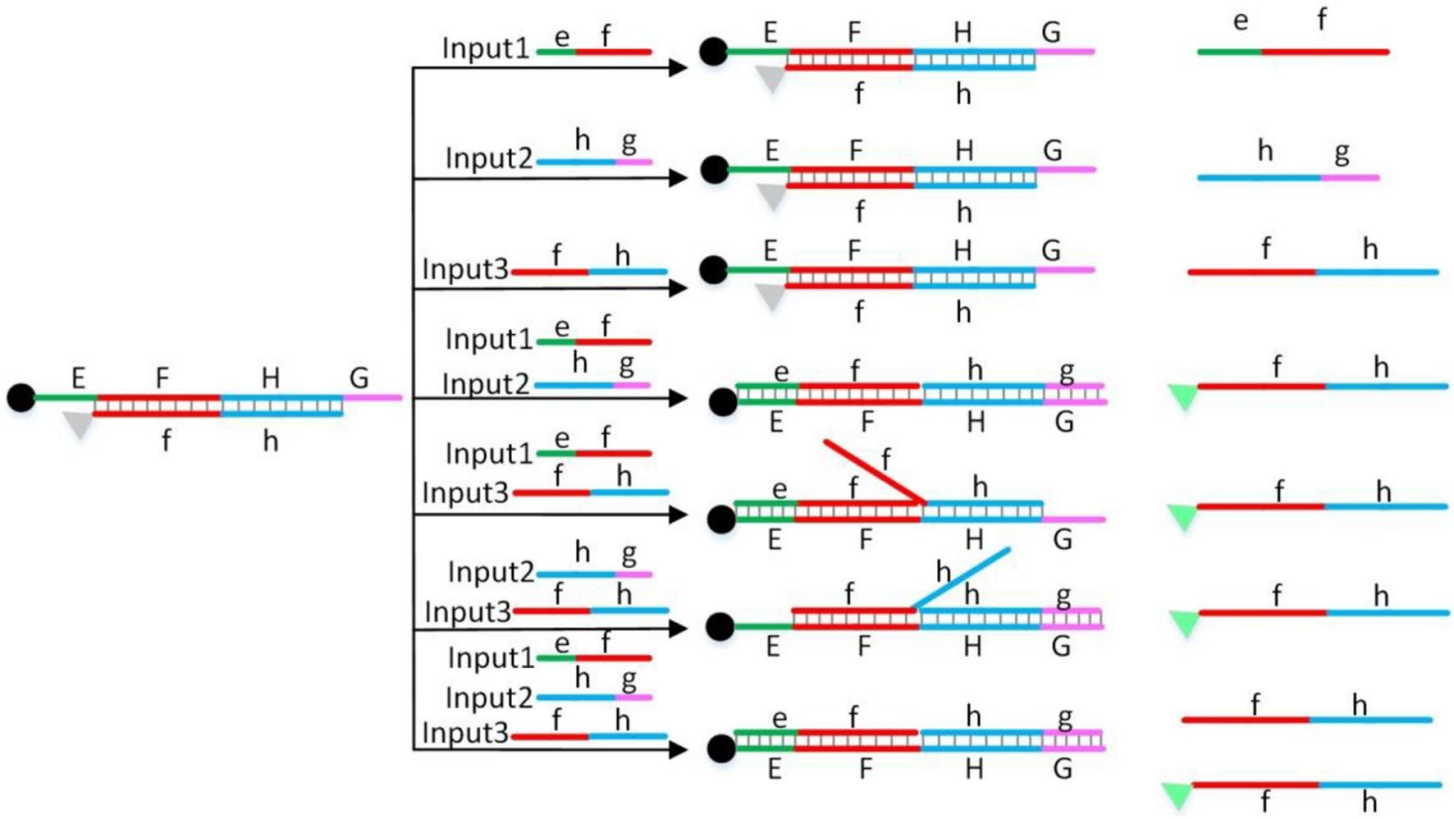
Three-input MAJORITY logic gate.

Figure 3 shows the principle of the three-input MAJORITY logic gate based on DNA strand displacement reaction. In the initial state, that is, without any input, the 3-terminal modified BHQ1 single DNA strand EFHG can hybridize with the 5-terminal modified FAM single DNA strand fh to form a double helix. The fluorescence signal of FAM is quenched due to the close distance between BHQ1 and FAM fluorescent group. As shown in Fig. 3, I designed three kinds of DNA input strands respectively: INPUT1, INPUT2, INPUT3. INPUT1 is ef, INPUT2 is hg, INPUT3 is fh. According to the principle of DNA strand displacement reaction, when any one of the three input strands is added alone, the input strand can not displace the fh strand, so the EFHG strand and fh strand are still hybridized, and the fluorescence signal of FAM is still quenched. Only when any two or three of the three input strands are added at the same time, the input strands can displace fh and hybridize with EFHG to release fluorescent group labeled fh strand. After the fh strand is released, its fluorescent group is far away from the quencher, so its fluorescent signal will increase. As shown in Fig. 3, a three-input MAJORITY logic gate is formed with three input strands ef, hg and fh as input and fluorescence signal as output.

### Experimental methods

The logic gates designed in this paper were verified by corresponding biochemical experiments. The specific experimental methods include the following three steps:

The first step, annealing: the DNA strands in the initial state was hybridized in 0.5 × TBE and 50 mM NaCl buffer, and the concentration of each DNA strand was 2 μm, the annealing temperature was room temperature.

The second step, adding Input strands for the strand displacement reaction: the annealed product was divided into equal samples, and the corresponding Input strands were added into the annealed product, respectively. The concentration ratio of Input strands to DNA at the annealing step was 1:1.

The third step, measure the fluorescence value: take out solutions with a DNA molar mass of 20 pmol from the solutions after strand displacement reaction respectively, then add the mixture of 0.5 × TBE and 50 mm NaCl into the solutions to the volume of 200 μl, and detect the fluorescence signals at the excitation wavelength of 495 nm and the emission wavelength of 520 nm with a fluorescence spectrophotometer.

### Experimental materials

All kinds of materials for the experiment were purchased from biological companies. The sequences of DNA strands used in the experiment are shown in Table 1 (the sequence color corresponds to the sequence color in Figs. 1, 2 and 3).

**Table 1:**
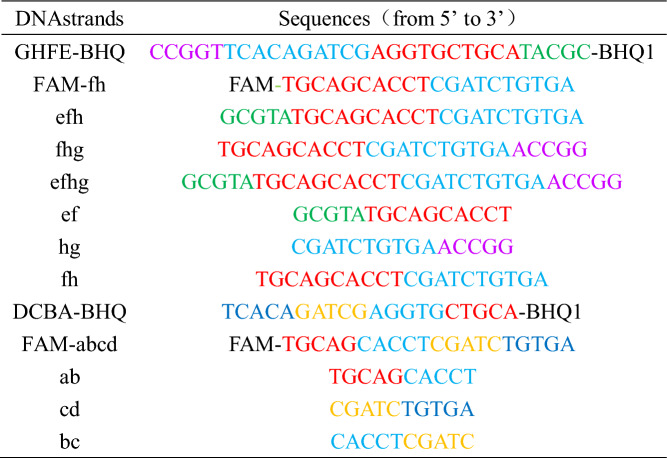
DNA sequences used in the experiment.

## Results and discussion

**Figure 4:**
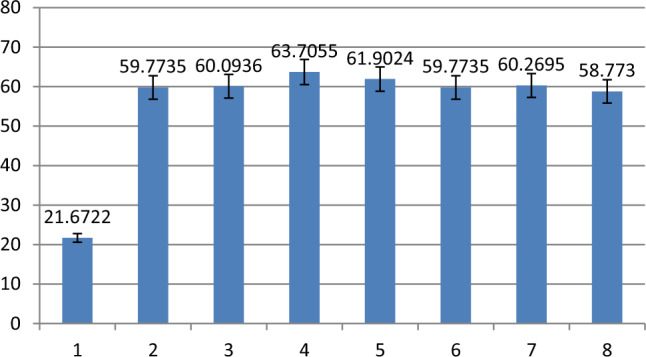
Florescence result of OR logic gate.

**Figure 5:**
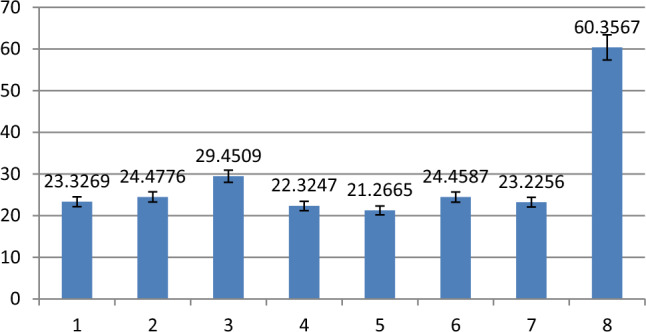
Florescence result of AND logic gate.

**Figure 6:**
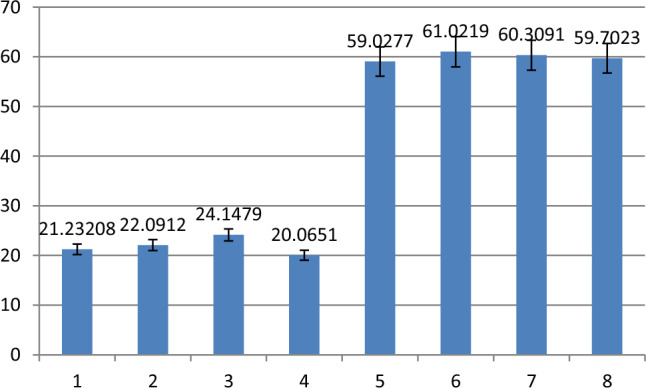
Florescence result of MAJORITY logic gate.

Figures 4, 5 and 6 shows the results of FAM fluorescence signal detected by fluorescence spectrophotometer after strand displacement reaction. Figures 4, 5 and 6 shows the histogram of fluorescence signal, the ordinate is the fluorescence signal value, the abscissa corresponds to the eight states of three-input logic gate: (0 0 0) (1 0 0) (0 1 0) (0 0 1) (1 1 0) (1 0 1) (0 1 1) (1 1 1). Taking 50 as the boundary, the fluorescence signals above 50 are defined as 1, and those below 50 are defined as 0. Table 2 shows the truth tables of three-input OR AND MAJORITY logic gates.

**Table 2:** The truth tables of three-input OR AND MAJORITY logic gates.

OR				AND				Majority			
Input1	Input2	Input3	Output	Input1	Input2	Input3	Output	Input1	Input2	Input3	Output
0	0	0	0	0	0	0	0	0	0	0	0
1	0	0	1	1	0	0	0	1	0	0	0
0	1	0	1	0	1	0	0	0	1	0	0
0	0	1	1	0	0	1	0	0	0	1	0
1	1	0	1	1	1	0	0	1	1	0	1
1	0	1	1	1	0	1	0	1	0	1	1
0	1	1	1	0	1	1	0	0	1	1	1
1	1	1	1	1	1	1	1	1	1	1	1

It can be seen from the histogram in Fig. 4 that the fluorescence signals of the seven states (1 0 0) (0 1 0) (0 0 1) (1 1 0) (1 0 1) (0 1 1) (1 1 1) are higher than the state (0 0 0). The fluorescence signal values of FAM in different input states conform to the results of three-input OR logic gate.

In Fig. 5, it can be seen from the histogram that the fluorescence signal of the state (1 1 1) is higher than the states (0 0 0) (1 0 0) (0 1 0) (0 0 1) (1 1 0) (1 0 1) (0 1 1). The fluorescence signal values of FAM in different input states conform to the results of three-input AND logic gate.

In Fig. 6, it can be seen from the histogram that the fluorescence signals of the states (1 1 0) (1 0 1) (0 1 1) (1 1 1) are higher than the states (0 0 0) (1 0 0) (0 1 0) (0 0 1). The fluorescence signal values of FAM in different input states conform to the results of three-input MAJORITY logic gate.

In summary, the experimental results show that DNA strand displacement technology has important application value in DNA computing, especially in the construction of DNA molecular logic gates. With the rapid development of Molecular Biology and Nanotechnology, DNA strand displacement technology combined with other new technologies will be more and more widely used in DNA computing and other fields. On the basis of these new technologies, DNA computing is bound to make breakthroughs in several development directions.

## Data Availability

The data is available at https://www.scidb.cn/s/Y3euMb in Science Date Bank.
